# Inception U-Net for Enhanced Breast Ultrasound Image Segmentation Using Transfer Learning

**DOI:** 10.3390/bioengineering13020181

**Published:** 2026-02-04

**Authors:** Yeonhyo Choi, Myoung Nam Kim, Sungdae Na

**Affiliations:** 1 Department of Medical & Biological Engineering, Graduate School, Kyungpook National University, Daegu 41404, Republic of Korea; yhchoi419@naver.com; 2 Department of Biomedical Engineering, School of Medicine, Kyungpook National University, Daegu 41404, Republic of Korea; kimmn@knu.ac.kr; 3 Department of Biomedical Engineering, Kyungpook National University Hospital, Daegu 41944, Republic of Korea

**Keywords:** breast ultrasound, image segmentation, deep learning, U-Net, inception, transfer learning, medical imaging

## Abstract

**Background**: Breast cancer diagnosis increasingly relies on ultrasound imaging, but challenges related to operator dependency and image quality limitations necessitate automated segmentation approaches. Traditional U-Net architectures, while widely used for medical image segmentation, suffer from shallow encoder structures that limit feature extraction capabilities. **Methods**: This study proposes an enhanced segmentation model that replaces the conventional U-Net encoder with an Inception architecture and employs transfer learning using ImageNet pre-trained weights. The model was trained and evaluated on a dataset of 900 breast ultrasound images from Kyungpook National University Hospital. Performance evaluation utilized multiple metrics including Intersection over Union (IoU), Dice coefficient, precision, and recall scores. **Results**: The proposed Inception U-Net achieved superior performance with an IoU score of 0.7774, Dice score of 0.8491, precision score of 0.7081, and recall score of 0.7174, demonstrating approximately 5% improvement over baseline U-Net architecture across all evaluation metrics. **Conclusions**: The integration of Inception modules within the U-Net architecture effectively addresses feature extraction limitations in breast ultrasound segmentation. Transfer learning from ImageNet datasets proves beneficial even across domain differences, establishing a foundation for broader medical imaging applications.

## 1. Introduction

Breast cancer remains one of the most prevalent malignancies affecting women globally, with increasing incidence rates observed in numerous other countries. This trend has correspondingly elevated the utilization of breast ultrasound imaging as a primary diagnostic modality due to its non-invasive nature, real-time imaging capabilities, and absence of ionizing radiation exposure. However, ultrasound imaging presents inherent challenges that can impact diagnostic accuracy and consistency [1,2,3,4,5].

The interpretation of breast ultrasound images is significantly influenced by operator expertise and experience levels, leading to potential variability in diagnostic outcomes. Additionally, ultrasound imaging typically exhibits lower spatial resolution compared to other medical imaging modalities such as magnetic resonance imaging (MRI) or computed tomography (CT), which can complicate the precise delineation of lesion boundaries. These limitations have motivated the development of computer-aided diagnosis (CAD) systems that leverage advanced image processing and machine learning techniques to enhance diagnostic accuracy and reduce inter-observer variability [6,7,8].

Deep learning approaches, particularly convolutional neural networks (CNNs), have demonstrated remarkable success in medical image analysis tasks, including segmentation, classification, and detection. Among various deep learning architectures, U-Net has emerged as the predominant choice for medical image segmentation due to its encoder–decoder structure with skip connections that preserve spatial information throughout the network. The architecture’s ability to combine low-level and high-level features makes it particularly suitable for pixel-level segmentation tasks in medical imaging applications [9,10].

Despite the widespread adoption of U-Net in medical image segmentation, the conventional architecture possesses inherent limitations that can impact segmentation performance. The standard U-Net encoder relies on relatively shallow convolutional and pooling operations, which may inadequately capture the complex multi-scale features present in medical images. This limitation is particularly pronounced in ultrasound imaging, where tissue boundaries often exhibit varying levels of contrast and clarity across different scales [11,12].

Transfer learning has emerged as a powerful technique for addressing data scarcity challenges in medical imaging applications. By leveraging pre-trained weights from large-scale datasets such as ImageNet, transfer learning enables models to benefit from learned feature representations that can be fine-tuned for specific medical imaging tasks. This approach is particularly valuable in medical imaging, where acquiring large annotated datasets is often challenging due to privacy concerns, expert annotation requirements, and data collection costs [13,14].

The objective of this study is to develop an enhanced U-Net architecture that overcomes the feature extraction limitations of conventional U-Net models for breast ultrasound image segmentation. We propose replacing the standard U-Net encoder with an Inception architecture and employing transfer learning strategies to improve segmentation performance. The study aims to demonstrate the effectiveness of this approach through comprehensive experimental validation and performance comparison with existing methods. The main contributions of this study can be summarized as follows:

- We propose an Inception-based U-Net architecture for breast ultrasound image segmentation to enhance multi-scale feature extraction capability within the encoder.
- We incorporate transfer learning using ImageNet pre-trained weights to mitigate the limitations associated with the relatively small size of annotated medical ultrasound datasets.
- We evaluate the proposed approach using standard segmentation metrics, including Intersection over Union (IoU) and Dice coefficient, and compare its performance with multiple encoder backbone-based U-Net variants.

The remainder of this paper is organized as follows. Section 2 reviews related work on medical image segmentation and transfer learning, with a focus on ultrasound imaging. Section 3 describes the dataset, preprocessing procedures, and the proposed Inception U-Net architecture. Section 4 presents the experimental results and comparative performance analysis. Section 5 discusses the clinical implications and limitations of the proposed method, and concludes the paper.

In addition to these variants, several studies have explored hybrid U-Net architectures incorporating multi-scale feature extraction modules, such as Inception-style encoders, to improve segmentation performance in complex medical imaging scenarios. These approaches highlight the importance of multi-scale contextual representation, particularly for images with heterogeneous textures [15,16,17].

## 2. Related Work

### 2.1. Medical Image Segmentation

Medical image segmentation has evolved significantly with the advent of deep learning technologies. Traditional approaches relied on threshold-based methods, region growing algorithms, and classical machine learning techniques, which often required extensive manual parameter tuning and domain-specific feature engineering. The introduction of fully convolutional networks (FCNs) marked a paradigm shift toward end-to-end learning approaches that could automatically learn relevant features for segmentation tasks.

U-Net architecture, originally proposed by Ronneberger et al., has become the de facto standard for biomedical image segmentation due to its effective handling of limited training data and precise localization capabilities. The architecture’s symmetric encoder–decoder structure with skip connections enables the preservation of spatial information while progressively extracting hierarchical features. Numerous variants and improvements to the original U-Net have been proposed, including attention mechanisms, residual connections, and dense connectivity patterns.

### 2.2. Transfer Learning in Medical Imaging

Transfer learning has proven particularly valuable in medical imaging applications where labeled datasets are often limited in size compared to natural image datasets. The technique involves leveraging knowledge learned from source domains (typically large-scale datasets like ImageNet) and adapting it to target domains (medical imaging tasks). Despite domain differences between natural and medical images, transfer learning has consistently demonstrated improved performance across various medical imaging applications.

Recent studies have investigated the effectiveness of different pre-trained architectures for medical image analysis, including VGG, ResNet, DenseNet, and EfficientNet. These investigations have revealed that the choice of pre-trained architecture can significantly impact final performance, with different architectures exhibiting varying degrees of transferability to medical imaging domains [18,19,20,21,22,23]. Furthermore, the concept of leveraging diverse feature representations has gained significant traction in other domains, such as lightweight bilateral networks for industrial defect detection and multi-task learning frameworks for complex identity and time estimation tasks. These multimodal and multi-task approaches provide valuable inspiration for enhancing the robustness of medical image segmentation by effectively separating and integrating task-specific features [24,25,26].

### 2.3. Breast Ultrasound Image Analysis

Breast ultrasound image analysis presents unique challenges due to the inherent characteristics of ultrasound imaging, including speckle noise, acoustic shadowing, and operator-dependent image quality variations. Previous research in automated breast ultrasound analysis has focused on lesion detection, classification, and segmentation tasks using various machine learning and deep learning approaches.

Convolutional neural networks have shown promising results for breast ultrasound image analysis, with studies demonstrating improved diagnostic accuracy and reduced inter-observer variability. However, most existing approaches rely on conventional CNN architectures without specifically addressing the multi-scale nature of features present in breast ultrasound images [27,28,29,30,31,32].

## 3. Materials and Methods

### 3.1. Dataset

The dataset utilized in this study comprises 900 breast ultrasound images obtained from Kyungpook National University Hospital. The dataset includes both original ultrasound images and corresponding ground truth segmentation masks annotated by experienced radiologists. The images were acquired using standard clinical ultrasound systems and represent diverse cases including various lesion types, sizes, and imaging conditions.

**Figure 1:**
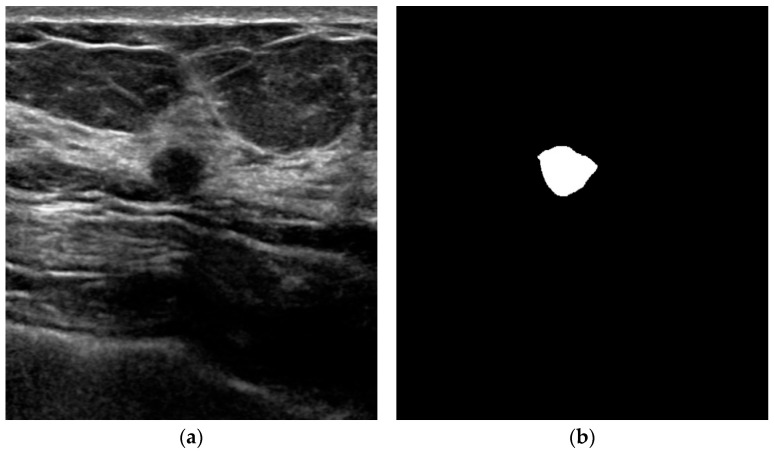
Learning data image for breast cancer lesion segmentation, (**a**) Breast cancer ultrasound image, (**b**) Breast cancer ultrasound ground truth.

Data preprocessing was performed using MATLAB (WathWorks, USA) environment to enhance image contrast and standardize image dimensions. The dataset was partitioned into training and testing sets, with 810 images allocated for training and 90 images reserved for testing purposes. This division ensures sufficient training data while maintaining an independent test set for objective performance evaluation [33]. Figure 1 shows a portion of the training data used. Figure 1a is an actual breast cancer image from an ultrasound scan, while Figure 1b is the segmented breast cancer image corresponding to the ground truth.

Preprocessing steps included image resizing to a fixed input resolution, intensity normalization, and contrast enhancement to reduce inter-scan variability. These preprocessing procedures contributed to stable model training and improved convergence behavior across different network architectures.

### 3.2. Proposed Architecture

**Figure 2:**
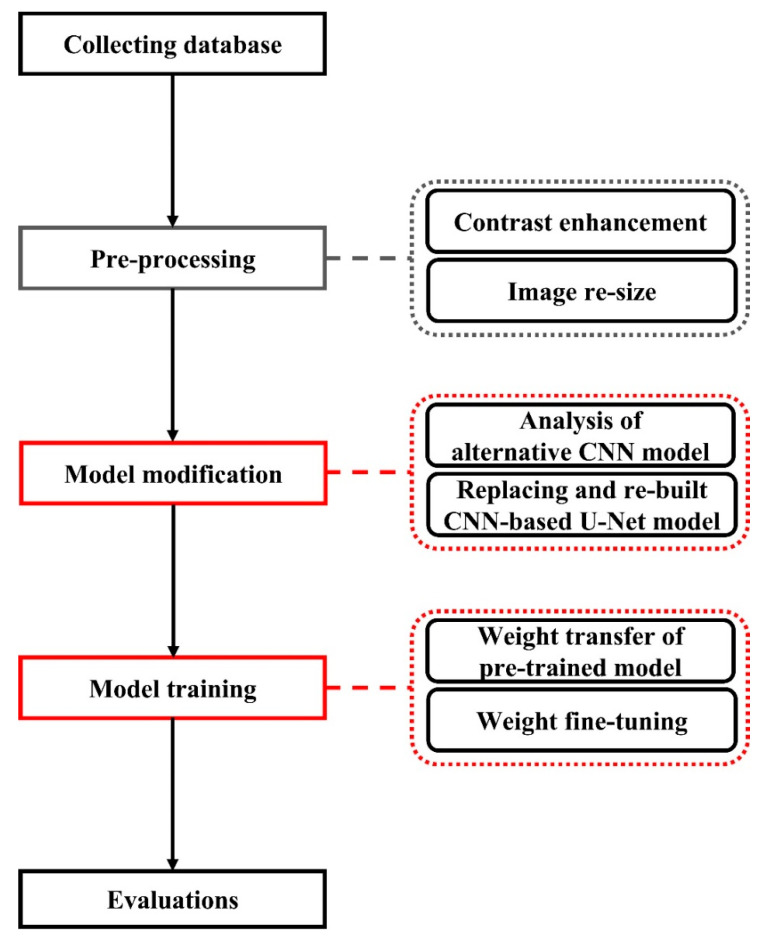
The flowchart of the proposed method.

The proposed architecture modifies the conventional U-Net structure by replacing the encoder with an Inception module-based architecture. The Inception modules enable multi-scale feature extraction through parallel convolutional operations with different kernel sizes, providing enhanced capability for capturing diverse feature representations within breast ultrasound images. Figure 2 is a schematic diagram of the final model of the proposed architecture. As can be seen in the diagram, a structure for parallel processing has been added to improve accuracy, as indicated by the blocks marked in red.

**Figure 3:**
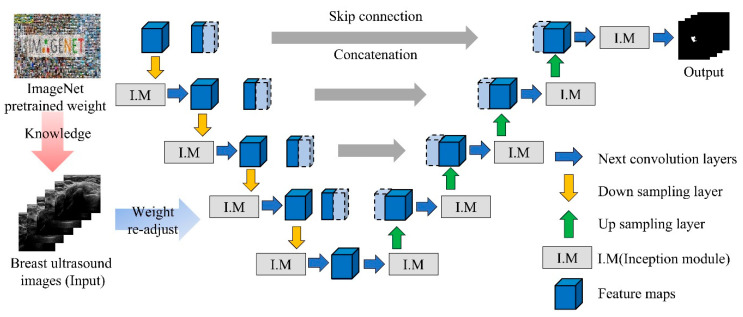
Inception-U-Net model based on transfer learning.

The encoder structure incorporates multiple Inception blocks that process input features through parallel pathways with 1 × 1, 3 × 3, and 5 × 5 convolutional filters, along with max-pooling operations. This multi-scale processing enables the network to capture both fine-grained details and broader contextual information simultaneously. The decoder structure maintains the traditional U-Net design with upsampling operations and skip connections that combine encoder features with decoder features at corresponding resolution levels. Figure 3 depicts the modified U-net-based architecture incorporating the proposed method.

### 3.3. Transfer Learning Implementation

Transfer learning was implemented by initializing the encoder weights with pre-trained ImageNet weights from the Inception model. Despite domain differences between natural images and medical ultrasound images, the low-level features learned from ImageNet provide a beneficial starting point for medical image analysis tasks.

Fine-tuning was performed by retraining the entire network on the breast ultrasound dataset while maintaining the pre-trained weight initialization. This approach allows the model to adapt the generic feature representations to the specific characteristics of ultrasound imaging while preserving beneficial knowledge from the pre-training phase.

### 3.4. Training Configuration and Evaluation Metrics

All experiments were conducted on a system equipped with Intel(R) Core(TM) i7-8700 CPU @ 3.20 GHz, 16 GB RAM, and GeForce GTX 1060 6 GB GPU. The implementation utilized Python programming language with PyTorch and TensorFlow frameworks. Data preprocessing was performed in MATLAB environment.

Training hyperparameters were configured as follows: batch size of 4, learning rate of 0.001, and 100 training epochs. The ReLU activation function was applied in hidden layers, while Softmax activation was used in the final segmentation module. The Adam optimizer was employed for parameter optimization.

Performance evaluation employed multiple standard metrics for segmentation tasks:

**Intersection over Union (IoU)**: Measures overlap between predicted and ground truth regions

**Dice Coefficient**: Evaluates spatial overlap similarity

**Precision**: Assesses positive prediction accuracy

**Recall**: Measures sensitivity in detecting true positive regions

## 4. Results and Discussion

### 4.1. Implementation Environment

Table 1 presents the system specifications and hyperparameters utilized in this study. All experiments were conducted under identical conditions to ensure fair comparison between different architectural approaches.

### 4.2. Segmentation Performance Comparison

Comparative evaluation was performed between the proposed Inception U-Net and several baseline architectures including conventional U-Net, VGGNet-U-Net, ResNet-U-Net, DenseNet-U-Net, and EfficientNet-U-Net. All models were trained using identical hyperparameters and evaluated on the same test dataset of 90 breast ultrasound images.

As summarized in Table 2, the proposed Inception U-Net achieved an IoU score of 0.7774 and a Dice coefficient of 0.8491, representing approximately a 5% improvement over the conventional U-Net baseline. Improvements were also observed in precision and recall metrics, indicating enhanced segmentation reliability.

**Figure 4:**
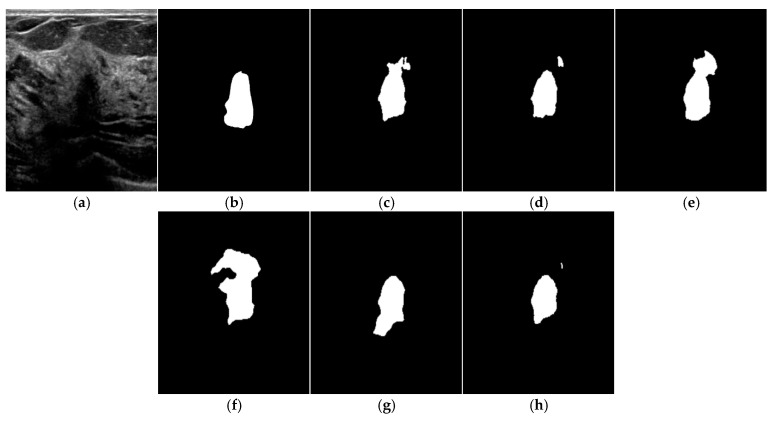
Test image result of each model, (**a**) Input image, (**b**) Label image, (**c**) Existing U-Net, (**d**) VGGNet-U-Net, (**e**) ResNet-U-Net, (**f**) DenseNet-U-Net, (**g**) EfficientNet-U-Net, (**h**) Inception-U-Net.

As illustrated in Figure 4, the proposed Inception U-Net demonstrates improved segmentation quality compared to baseline architectures.

### 4.3. Qualitative Analysis

Figure 4 illustrates representative segmentation results produced by each model. Visual inspection indicates that the proposed Inception U-Net achieves more accurate lesion boundary delineation compared to baseline approaches. In particular, the multi-scale feature extraction capability of the Inception-based encoder enables improved segmentation performance in cases involving irregular lesion shapes and heterogeneous contrast levels.

The proposed method demonstrates improved robustness in challenging imaging conditions, such as low-contrast regions and partial acoustic shadowing, where conventional U-Net-based approaches tend to produce fragmented or incomplete segmentation results.

Although visual differences may appear subtle, improved boundary consistency and reduced segmentation irregularities are clinically meaningful for lesion size estimation and longitudinal follow-up in breast ultrasound examinations.

### 4.4. Transfer Learning Effectiveness

**Figure 5:**
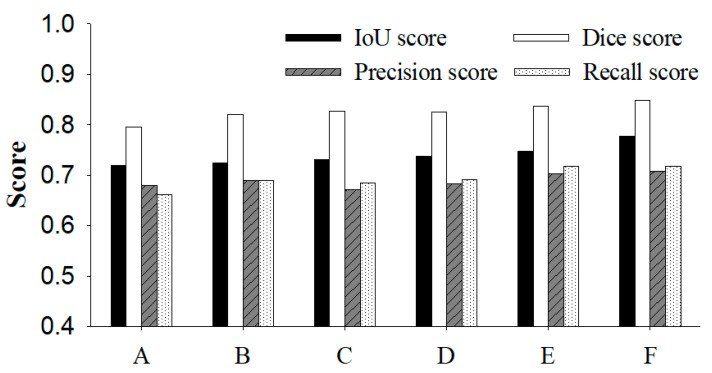
Quantitative comparison of segmentation performance across different models, (A) U-Net, (B) VGG-U-Net, (C) ResNet-U-Net, (D) DenseNet-U-Net, (E) Efficient-U-Net, and (F) proposed method.

The observed performance improvements can be partly attributed to the effective use of transfer learning with ImageNet pre-trained weights. Despite the domain gap between natural images and medical ultrasound images, low-level features learned from large-scale datasets provide a strong initialization that accelerates convergence and enhances final segmentation performance. Figure 5 presents results from various models for evaluation metrics used in image segmentation. Compared to the various existing models (A–E), the results from the proposed model (F) demonstrate significant improvements.

Fine-tuning enables the model to adapt these generic feature representations to ultrasound-specific image characteristics while preserving beneficial knowledge from pre-training. This approach effectively addresses the challenge of limited annotated medical imaging datasets.

### 4.5. Comparison with Exising Studies

To facilitate objective positioning of the proposed method within existing research, a literature-based quantitative comparison using standardized segmentation metrics, including the Dice coefficient and Intersection over Union (IoU), is provided in Table 3. Although direct re-implementation of all recent state-of-the-art models on public datasets was not feasible, Table 3 summarizes representative performance values reported in prior breast ultrasound segmentation studies.

Despite differences in datasets and experimental settings, the proposed method demonstrates competitive performance relative to existing approaches, supporting the effectiveness of integrating an Inception-based encoder with transfer learning for breast ultrasound image segmentation.

### 4.6. Discussion on Clinical Scope and Architectural Rationale

It should be noted that the primary objective of this study is to enhance segmentation accuracy for clinical decision support rather than to optimize real-time inference or deployment on resource-constrained platforms. Accordingly, the proposed Inception-based U-Net prioritizes improved feature representation and segmentation reliability and is intended for offline or near–real-time clinical analysis.

From an architectural perspective, the advantage of the Inception-based encoder lies in its ability to capture multi-scale contextual information through parallel convolutional pathways with different receptive fields. This property is particularly relevant for ultrasound imaging, where lesion boundaries often exhibit heterogeneous textures and scale-dependent contrast variations. By aggregating features at multiple spatial scales, the Inception module enhances the encoder’s capacity to represent complex anatomical patterns compared to the conventional U-Net encoder.

### 4.7. Theoretical Analysis of Convergence and Generalization

The convergence and generalization capabilities of the proposed Inception U-Net are theoretically grounded in its architectural design and optimization strategy.

First, regarding convergence, initializing the encoder with ImageNet pre-trained weights provides a ‘warm-start’ in the optimization landscape. This places the initial parameters in a region where low-level features (e.g., edges and textures) are already well-represented, facilitating faster and more stable convergence compared to random initialization. Furthermore, the skip-connection mechanism inherent in the U-Net framework ensures effective gradient flow throughout the network, mitigating the vanishing gradient problem and ensuring consistent convergence during fine-tuning.

Second, regarding generalization, the integration of Inception modules serves as a form of structural regularization. By utilizing parallel convolutional pathways with varying kernel sizes (1 × 1, 3 × 3, 5 × 5), the model captures multi-scale contextual information simultaneously. This multi-scale adaptation allows the network to be less sensitive to variances in lesion size and shape, thereby reducing the risk of over-fitting to scale-specific patterns in a single-center dataset. The diversity of the learned feature space, combined with the regularization effect of transfer learning, collectively strengthens the model’s ability to generalize to unseen ultrasound images despite the inherent noise and artifacts.

## 5. Conclusions and Limitations

The increasing incidence of breast cancer among women in Korea and several other countries has led to a corresponding rise in breast ultrasound imaging for diagnostic purposes. However, ultrasound imaging presents inherent challenges, including dependency on operator expertise and relatively lower image resolution compared to other medical imaging modalities, which can complicate accurate diagnosis. To address these limitations, deep learning-based lesion segmentation models have emerged as promising complementary technologies.

The accuracy of deep learning-based medical image segmentation models is primarily determined by effective feature extraction and learning from diverse image characteristics, as well as precise transmission of positional information for lesion regions. While U-Net models have been widely adopted for segmentation tasks in medical imaging, including organ and lesion segmentation, traditional U-Net architectures suffer from limitations in their encoder structure, characterized by shallow convolutional and pooling operations that can lead to inadequate feature extraction and pixel information loss.

In this study, we addressed these limitations by implementing an encoder structure improvement that significantly enhances model performance. Based on experimental results, we selected the Inception model to replace the conventional U-Net encoder architecture. To compensate for limited training data availability, we employed transfer learning by utilizing pre-trained weights from large-scale ImageNet datasets and subsequently applied fine-tuning through additional retraining to capture ultrasound-specific image characteristics.

Our comprehensive experimental evaluation demonstrates the superiority of the proposed Inception U-Net architecture across multiple performance metrics. The model achieved an IoU score of 0.7774, Dice score of 0.8491, precision score of 0.7081, and recall score of 0.7174, representing approximately 5% improvement over the baseline U-Net model. These results validate that the Inception model structure provides more effective feature extraction and learning capabilities for ultrasound image analysis compared to traditional U-Net encoder architectures. Furthermore, the successful application of ImageNet pre-trained weights, despite the domain difference between natural and medical images, demonstrates the positive impact of transfer learning in medical image segmentation tasks.

The clinical significance of this research extends beyond technical improvements in segmentation accuracy. The proposed method enables effective lesion region segmentation in breast ultrasound images, potentially serving as a diagnostic support system to assist medical professionals in clinical decision-making. While this study focused specifically on ultrasound imaging within a constrained scope, the transfer learning techniques and architectural improvements developed here establish a foundation for broader applications across diverse medical imaging modalities.

The present study does not include a stratified analysis based on lesion pathology (benign versus malignant) or lesion size due to limited availability of balanced annotations. In addition, robustness under extreme ultrasound acquisition conditions, such as severe speckle noise, acoustic shadowing, and low-contrast imaging, was not quantitatively evaluated. These factors represent important considerations for real-world ultrasound applications and will be addressed in future studies using larger, more diverse datasets.

Future research directions include extending the developed transfer learning methodologies to various clinical domains and imaging modalities, including magnetic resonance imaging (MRI), computed tomography (CT), and positron emission tomography (PET). The core transfer learning technologies validated in this work are expected to contribute to the development of promising artificial intelligence solutions in the broader field of medical image analysis, ultimately advancing computer-aided diagnosis capabilities across multiple medical imaging applications.

The demonstrated effectiveness of combining advanced CNN architectures with transfer learning approaches provides a robust framework for addressing similar challenges in medical image segmentation, contributing to the ongoing evolution of AI-assisted healthcare technologies.

## Figures and Tables

**Table 1:** System Environment and Hyperparameters.

Category	Item	Contents
System	Processor	Intel(R) Core(TM) i7-8700 CPU @ 3.20 GHz
	OS	Windows 10, 64-bit
	GPU	GeForce GTX 1060, 6 GB
	Programming Language	Python 3.6, MATLAB 2021
	Framework	PyTorch, TensorFlow
Hyperparameters	Activation Function	ReLU, Softmax
	Optimization	Adam
	Batch Size	4
	Learning Rate	0.001
	Epochs	100

**Table 2:** Performance Evaluation Results for 90 Test Images.

Model	IoU Score	Dice Score	Precision Score	Recall Score
Existing U-Net	0.7196	0.7951	0.6798	-
VGGNet-U-Net	0.7239	0.8206	0.6895	-
ResNet-U-Net	0.7318	0.8266	0.6723	-
DenseNet-U-Net	0.7374	0.8249	0.6825	-
EfficientNet-U-Net	0.7474	0.8361	0.7036	-
Inception U-Net (Proposed)	0.7774	0.8491	0.7081	0.7174

**Table 3:** Performance Evaluation Results for other segmentation models.

Study	Year	Dataset	Method	Dice	IoU	Notes
Ronneberger et al. [4]	2015	Private	U-Net	0.79	0.72	Baseline
Zhuang et al. [5]	2019	BUSI	RDAU-Net	0.82	0.74	Residual + Dense
Zhou et al. [12]	2018	BUSI	U-Net++	0.83	0.75	Nested skip
Alom et al. [11]	2018	BUSI	R2U-Net	0.84	0.76	Recurrent
Tan & Le [19]	2020	BUSI	EfficientNet-U-Net	0.84	0.75	Scaled backbone
This study	2025	Private	Inception U-Net (TL)	0.849	0.777	Single-center

## Data Availability

The data presented in this study are available on request from the corresponding author. The data are not publicly available due to privacy and ethical restrictions related to medical imaging data.
